# Development of twist‐correction system for radiotherapy of head and neck cancer patients

**DOI:** 10.1002/acm2.12667

**Published:** 2019-06-20

**Authors:** Hidetoshi Shimizu, Koji Sasaki, Takahiro Aoyama, Shigeru Matsushima, Taiki Isomura, Hiroshi Fukuma, Hiroyuki Tachibana, Takeshi Kodaira

**Affiliations:** ^1^ Department of Radiation Oncology Aichi Cancer Center Hospital Aichi Japan; ^2^ Graduate School of Radiological Technology Gunma Prefectural College of Health Sciences Gunma Japan; ^3^ Department of Diagnostic and Interventional Radiology Aichi Cancer Center Hospital Aichi Japan; ^4^ Department of Radiology Nagoya City University Hospital Aichi Japan

**Keywords:** IMRT, head and neck, twist‐correction, 6DoF

## Abstract

To propose a concept for correcting the twist between the head and neck and the body frequently occurring in radiotherapy patients and to develop a prototype device for achieving this. Furthermore, the operational accuracy of this device under no load was evaluated. We devised a concept for correcting the twist of patients by adjustment of the three rotation (pitch, roll, and yaw) angles in two independent plates connected by a joint (fulcrum). The two plates (head and neck plate and body plate) rotate around the fulcrum by adjusting screws under each of them. A prototype device was created to materialize this concept. First, after all adjusting screws were set to the zero position, the rotation angle of each plate was measured by a digital goniometer. Repeatability was evaluated by performing 20 repeated measurements. Next, to confirm the rotational accuracy of each plate of the prototype device, the calculated rotation angles for 20 combinations of patterns of traveled distances of the adjusting screws were compared with those measured by the digital goniometer and cone‐beam computed tomography (CT). The repeatability (standard deviation: SD) of the pitch, roll, and yaw angles of the head and neck plate was 0.04°, 0.05°, and 0.03°, and the repeatability (SD) of the body plate was 0.05°, 0.04°, and 0.04°, respectively. The mean differences ± SD between the calculated and measured pitch, roll, and yaw angles for the head and neck plate with the digital goniometer were 0.00 ± 0.06°, −0.01 ± 0.06°, and −0.04 ± 0.04°, respectively. The differences for the body plate were −0.03 ± 0.04°, 0.03 ± 0.05°, and 0.02 ± 0.05°, respectively. Results of the cone‐beam CT were similar to those of the digital goniometer. The prototype device exhibited good performance regarding the rotational accuracy and repeatability under no load. The clinical implementation of this concept is expected to reduce the residual error of the patient position due to the twist.

## 1 INTRODUCTION

Intensity‐modulated radiotherapy (IMRT) can deliver the dose to tumor lesions locally and reduce the dose delivered to the normal tissue surrounding the tumor. Dose escalation to the tumor by this technique can improve the treatment outcome. In particular, IMRT for cases of head and neck (HN) cancer can deliver adequate doses to complex‐shaped tumors surrounding normal tissues, such as the spinal cord and brain stem. In addition, IMRT can reduce the dose to the parotid glands, submandibular glands, and oral cavity; therefore, it alleviates salivary disorder and improves the patient's quality of life. On the other hand, to maximize these advantages of IMRT, it is important to use an image‐guided system to match the patient's position in the radiotherapy with that in the treatment planning computed tomography (CT). However, we often experience misalignment of the tumor shape due to twisting of the patient's neck. Correction by the current image‐guided system can be applied only to six axes (three translation axes and three rotation axes) to the whole body of the patient,^1^, ^2^, ^3^ so the partial twist of the patient cannot be completely canceled.^4^, ^5^, ^6^, ^7^ This results in errors remaining even after correction. Such potential error is compensated for by adding a safety margin around the tumor; however, this approach interferes with the benefits of IMRT, which can deliver the dose to tumor lesions locally. The aim of this study was to propose a concept for correcting the above‐mentioned twist of the patient and to develop a prototype device for achieving this. Furthermore, the operational accuracy of this device under no load was evaluated.

## 2 MATERIALS AND METHODS

### 2.1 Schema of the prototype

**Figure 1:**
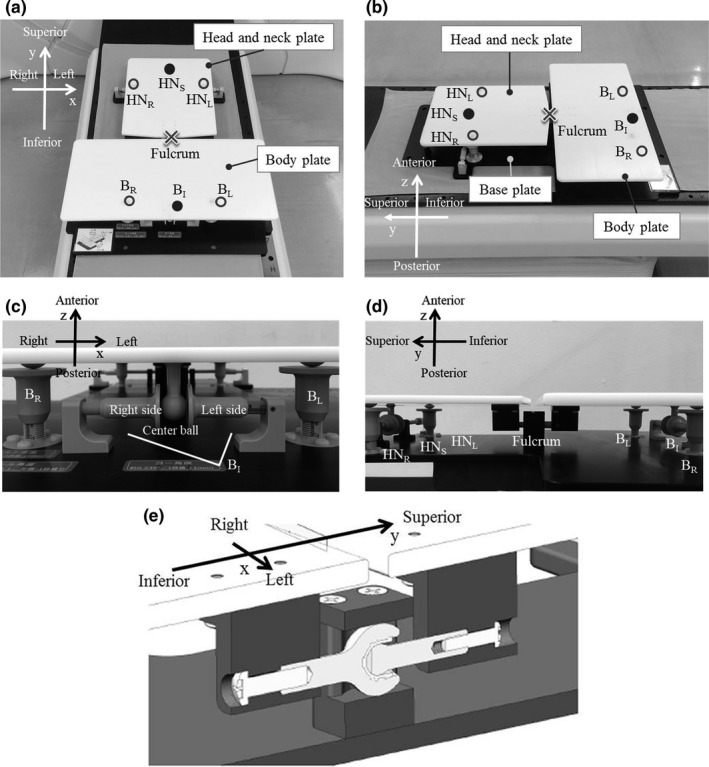
The prototype of the twist‐correction device: (a) and (b) the positions of the angle‐adjusting screws (HN_S_, HN_R_, HN_L_, B_R_, B_L_, and B_I_), (c) an inferior‐side view, (d) a right‐side view, and (e) the joint (fulcrum) of the head and neck (HN) plate and the body plate

**Figure 2:**
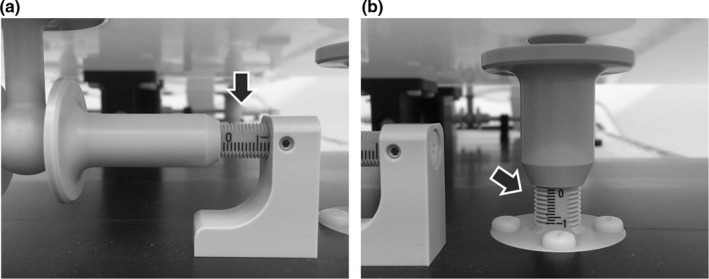
Enlarged image of the two types of adjusting screws: (a) and (b). The arrows show the scale on the surface of the screw

We devised a concept that can correct the twist of the patient by adjustment of the three rotation angles in two independent plates connected by a joint (fulcrum). Figure 1 shows the prototype device made to materialize this concept. As illustrated in Fig. 1(a) and (b), two plates (HN plate and body plate) of different sizes upon the base plate were connected by a fulcrum. The HN and body plates rotate around the fulcrum in three axes, right–left (x), inferior–superior (y), and anterior–posterior (z) axes, by adjusting screws under the plates. The circles in Fig. 1(a) and (b) show the positions of the angle‐adjusting screws (HN_S_, HN_R_, HN_L_, B_R_, B_L_, and B_I_). The screws marked with the filled circles and empty circles move in the x and z directions, respectively. Figure 1(c) and (d) show the inferior‐side view and the right‐side view of the device, respectively. B_I_ consists of the center ball and the left and right screws [Fig. 1(c)]. While loosening the screw on the right side and tightening the screw on the left side, the ball moves in the left direction, and the body plate rotates around the fulcrum to the left (and vice versa). Figure 1(e) is a sectional view of the fulcrum shown in Fig. 1(d). The traveled distances of the adjusting screws can be confirmed because the scale is engraved on the surface of the adjusting screws, as shown by the arrow in Fig. 2. The material and density of the components of the prototype device are given in Table 1.

**Table 1:** Material and physical density of the components of the device

Component	Material	Physical density [g cm^−3^]
Head and neck plate	Polyoxymethylene (POM)	1.41
Body plate	Polyoxymethylene (POM)	1.41
Base Plate	Polyoxymethylene (POM)	1.41
Pan head screw	RENY	1.65
Flat head screw	RENY	1.65
The others	Polyether ether ketone (PEEK)	1.30

### 2.2 Theory of rotation angle calculation

**Figure 3:**
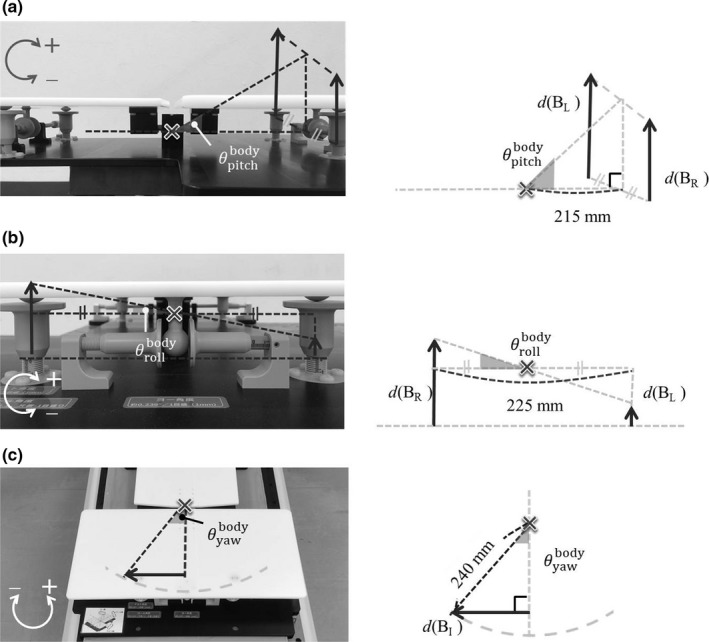
Relationship between the rotation angle and the traveled distance of the adjusting screw: (a) pitch, (b) roll, and (c) yaw angles

Each plate rotates around the fulcrum using three adjusting screws under the plate. Figure 3 shows the relationship between the rotation angle and the traveled distance of each adjusting screw. For example, the pitch and roll angles (the rotation angles for the x‐ and y‐axes, respectively) of the body plate can be obtained by adjusting the screws B_R_ and B_L_. The angles can be calculated by the traveled distances of the screws B_R_ and B_L_ [*d*(B_R_) and *d*(B_L_), respectively] in Fig. 3(a) and (b). Moreover, the yaw angle (the rotation angle for the z‐axis) can be calculated by the traveled distance of the screw B_I_ [*d*(B_I_)], as shown in Fig. 3(c). The rotation angle with respect to each translation axis (x, y, and z) of the plate is calculated by the traveled distance of the adjusting screw and the known distance between the adjusting screws as follows:

(1) $$\theta_{\text{pitch}}^{i}=\tan^{-1}|\frac{\frac{d\left(i_{\text{R}}\right)+d\left(i_{\text{L}}\right)}{2}}{215}|\left(-10\leq d\left(i_{\text{R}}\right)\leq 10,\;-10\leq d\left(i_{\text{L}}\right)\leq 10\right),$$

(2) $$\theta_{\text{roll}}^{i}=\tan^{-1}|\frac{d\left(i_{\text{R}}\right)-d\left(i_{\text{L}}\right)}{225}|\left(-10\leq d\left(i_{\text{R}}\right)\leq 10,\;-10\leq d\left(i_{\text{L}}\right)\leq 10\right),$$

(3) $$\theta_{\text{yaw}}^{i}=\sin^{-1}|\frac{d\left(i_{p}\right)}{240}|\left(-10\leq d\left(i_{p}\right)\leq 10\right),$$

where $\theta_{\text{pitch}}^{i}$, $\theta_{\text{roll}}^{i}$, and $\theta_{\text{yaw}}^{i}$ show the pitch, roll, and yaw angles of plate *i* (HN or body) and *d* indicates the traveled distance of the adjusting screw. When *i* is HN, *d*(*i_p_*) shows the traveled distance of the adjusting screw HN_S_, and when *i* is body, *d*(*i_p_*) shows that of the adjusting screw B_I_. The movable ranges of $\theta_{\text{pitch}}^{i}$, $\theta_{\text{roll}}^{i}$, and $\theta_{\text{yaw}}^{i}$ calculated from the traveled distance of the adjusting screw were from −2.66 to 2.66, −5.08 to 5.08, and −2.39 to 2.39, respectively. The clockwise direction for each translation axis was defined as positive angles.

### 2.3 Operational accuracy of the prototype device

First, after all adjusting screws were set to the zero position, the rotation angle of each plate was measured by a digital goniometer (DP–90; Niigata Seiki Co., Ltd.; detection limit: 0.05°). Repeatability was evaluated by 20 repeated measurements.

**Figure 4:**
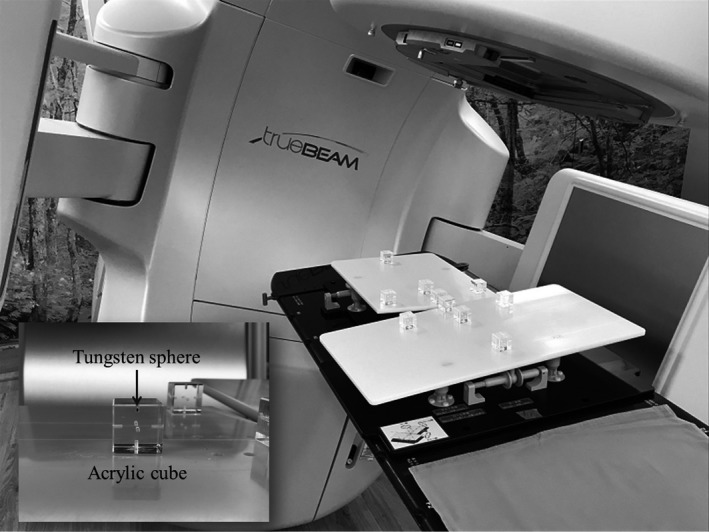
Photograph of the cubic phantoms installed to confirm the rotational accuracy of the prototype device by Varian cone‐beam computed tomography imaging system. The tungsten sphere of 1 mm at diameter was inserted into each acrylic cube

Next, 20 combinations of traveled distances of the six adjusting screws were obtained using random numbers from Microsoft Excel 2010 (Table 2). To confirm the rotational accuracy of the prototype device, $\theta_{\text{pitch}}^{i}$, $\theta_{\text{roll}}^{i}$, and $\theta_{\text{yaw}}^{i}$ calculated at a given traveled distance were compared with those measured by a digital goniometer. Since it was not possible to measure the yaw angle when the device was lying on the floor, this measurement was performed at a position of 90° to the floor. In addition, ten acrylic cubic phantoms with tungsten sphere of 1 mm in diameter were placed on the prototype device (Fig. 4), and after movement by 20 combinations of six adjustment screws, scanning was performed with a Varian cone‐beam CT imaging system. The resolutions of the cone‐beam CT images for x, y, and z directions were 0.5, 1.0, and 0.5 mm, respectively. The obtained cone‐beam CT image sets were imported into MIM maestro (MIM Software Inc., OH, USA), which was a commercial software system widely used for image registration. Then, the images before and after the movement were registered, and the rotational angles from the zero position of the HN plate and the body plate were obtained using a box‐based alignment tool in the MIM maestro software.

**Table 2:** Combination patterns for the traveled distances of the adjusting screws

Pattern	Traveled distance [mm]					
	HN_S_	HN_R_	HN_L_	B_R_	B_L_	B_I_
1	−9	−4	6	−4	3	−8
2	5	3	4	−10	−10	−9
3	−3	−9	−6	−10	−7	2
4	6	10	−6	−2	−9	2
5	4	−3	4	4	8	−1
6	−5	−6	5	−7	−10	−7
7	1	−5	10	6	2	9
8	5	1	9	−7	−8	3
9	−6	5	−2	4	6	−8
10	−7	0	−4	0	3	10
11	6	4	−10	−4	9	−4
12	−10	9	9	6	−4	−6
13	−3	5	−3	10	7	−2
14	6	1	10	4	−7	9
15	−4	2	−8	6	−8	6
16	7	5	3	−4	−7	−6
17	−7	−1	0	8	3	−2
18	−8	5	8	−4	−4	−6
19	3	−4	−9	−9	2	−5
20	3	2	−10	−3	1	−10
Maximum	7	10	10	10	9	10
Minimum	−10	−9	−10	−10	−10	−10

## 3 RESULTS

### 3.1 Evaluation of the repeatability

According to the 20 repeated measurements, the repeatability (standard deviation: SD) of $\theta_{\text{pitch}}^{\text{HN}}$, $\theta_{\text{roll}}^{\text{HN}}$, and $\theta_{\text{yaw}}^{\text{HN}}$ was 0.04°, 0.05°, and 0.03°, respectively. The repeatability (SD) of $\theta_{\text{pitch}}^{\text{body}}$, $\theta_{\text{roll}}^{\text{body}}$, and $\theta_{\text{yaw}}^{\text{body}}$ was 0.05°, 0.04°, and 0.04°, respectively. The repeatability was less than the detection limit of the digital goniometer (0.05°).

### 3.2 Evaluation of the rotational accuracy

**Figure 5:**
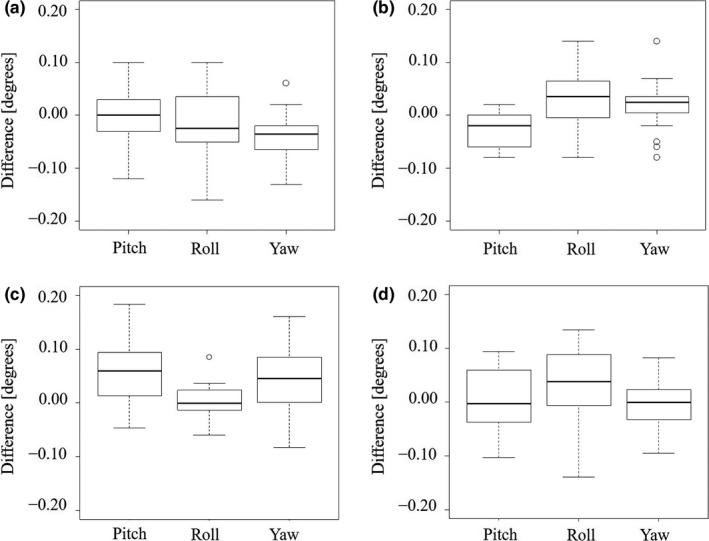
The differences between the calculated and measured rotation angles in the combinations of 20 patterns for the adjusting screws: (a) for the HN plate with the digital goniometer, (b) for the body plate with the digital goniometer, (c) for the HN plate with cone‐beam computed tomography, and (d) for the body plate with the cone‐beam computed tomography

Table 3 shows the values of $\theta_{\text{pitch}}^{i}$, $\theta_{\text{roll}}^{i}$, and $\theta_{\text{yaw}}^{i}$ calculated by the combination patterns for the traveled distances of the adjusting screws in Table 2. Figure 5 shows the differences between the calculated and measured rotation angles in the combinations of 20 patterns for the adjusting screws. The mean differences ± SD of $\theta_{\text{pitch}}^{\text{HN}}$, $\theta_{\text{roll}}^{\text{HN}}$, and $\theta_{\text{yaw}}^{\text{HN}}$ by digital goniometer were 0.00 ± 0.06°, −0.01 ± 0.06°, and −0.04 ± 0.04°, respectively. The differences of $\theta_{\text{pitch}}^{\text{body}}$, $\theta_{\text{roll}}^{\text{body}}$, and $\theta_{\text{yaw}}^{\text{body}}$ were −0.03 ± 0.04°, 0.03 ± 0.05°, and 0.02 ± 0.05°, respectively. The mean differences ± SD of $\theta_{\text{pitch}}^{\text{HN}}$, $\theta_{\text{roll}}^{\text{HN}}$, and $\theta_{\text{yaw}}^{\text{HN}}$ by cone‐beam CT were 0.06 ± 0.06°, 0.00 ± 0.03°, and 0.04 ± 0.06°, respectively. The differences of $\theta_{\text{pitch}}^{\text{body}}$, $\theta_{\text{roll}}^{\text{body}}$, and $\theta_{\text{yaw}}^{\text{body}}$ were 0.00 ± 0.06°, 0.04 ± 0.08°, and 0.00 ± 0.04°, respectively.

**Table 3:** Rotation angles calculated from the combination patterns for the traveled distances of the adjusting screws in Table 2

Pattern	Calculated angles [degrees]					
	$\theta_{\text{pitch}}^{\text{HN}}$	$\theta_{\text{roll}}^{\text{HN}}$	$\theta_{\text{yaw}}^{\text{HN}}$	$\theta_{\text{pitch}}^{\text{body}}$	$\theta_{\text{roll}}^{\text{body}}$	$\theta_{\text{yaw}}^{\text{body}}$
1	0.27	−2.54	−2.15	0.13	−1.78	−1.91
2	0.93	−0.25	1.19	2.66	0.00	−2.15
3	−2.00	−0.76	−0.72	2.26	−0.76	0.48
4	0.53	4.07	1.43	1.47	1.78	0.48
5	0.13	−1.78	0.95	−1.60	−1.02	−0.24
6	−0.13	−2.80	−1.19	2.26	0.76	−1.67
7	0.67	−3.81	0.24	−1.07	1.02	2.15
8	1.33	−2.04	1.19	2.00	0.25	0.72
9	0.40	1.78	−1.43	−1.33	−0.51	−1.91
10	−0.53	1.02	−1.67	−040	−0.76	2.39
11	−0.80	3.56	1.43	−0.67	−3.31	−0.95
12	2.40	0.00	−2.39	−0.27	2.54	−1.43
13	0.27	2.04	−0.72	−2.26	0.76	−0.48
14	1.47	−2.29	1.43	0.40	2.80	2.15
15	−0.80	2.54	−0.95	0.27	3.56	1.43
16	1.07	0.51	1.67	1.47	0.76	−1.43
17	−0.13	−0.25	−1.67	−1.47	1.27	−0.48
18	1.73	−0.76	−1.91	1.07	0.00	−1.43
19	−1.73	1.27	0.72	0.93	−2.80	−1.19
20	−1.07	3.05	0.72	0.27	−1.02	−2.39
Maximum	2.40	4.07	1.67	2.66	3.56	2.39
Minimum	−2.00	−3.81	−2.39	−2.26	−3.31	−2.39

## 4 DISCUSSION

In this paper, we propose a concept for correcting the twist that is frequently exhibited in the neck region of patients undergoing radiotherapy and developed a prototype device for embodying this concept. To the best of our knowledge, this is the first report of a device applied for correcting the twist of a patient. The prototype device might be able to reduce the residual error of the patient position due to this twist and reduce the additional margin needed for compensating the adequate dose to tumors.

Correction of the patient position by the current image‐guided system is mainly performed on six axes using the six degrees of freedom (6DoF) couch. There is also a treatment machine, called tomotherapy, which can correct only three translational and one rotational axes.^8^ Zhang et al. investigated the rotational accuracy of the 6DoF couch with cone‐beam CT,^9^ and they reported that the mean rotational errors ± SD for pitch, roll, and yaw angles were 0.028 ± 0.032°, −0.043 ± 0.058°, and −0.009 ± 0.033°, respectively. The rotational accuracy of our prototype device was measured using two different methods (the digital goniometer and cone‐beam CT), which is almost equivalent to the results reported by Zhang et al.^9^ as shown in Result section 3.B. Moreover, the repeatability was less than the detection limit of the digital goniometer, as described in Result section 3.A. The prototype device exhibited good performance regarding the rotational accuracy and the repeatability under no load.

For the systems excluding the 6DoF couch, Dhabaan et al. reported that pitch and roll angles can be corrected by mounting the customized device on the top of the treatment couch in the cranial position.^10^ Belcher et al. and Ostyn et al. developed an electromechanical robotic system that can provide precise 6DoF motion trajectories.^11^, ^12^ These systems can correct the rotational position of the patient by themselves; therefore, they might be able to correct the twist with the couch, which has the ability to achieve rotational correction. Our system has versatility because it can add twist‐correction to a radiotherapy device with a couch that can only correct translation movement.

The density of polyoxymethylene and polyether ether ketone materials in Table 1 was smaller than that of the cortical bone material (Gammex, Inc., density; 1.559 g cm^−3^). RENY with the nearest density to that of the cortical bone was used in the screw for connecting the parts of the prototype device, but the amount used in the prototype device was small. There is no influence on the target delineation because there are few artifacts in the CT images. If the dose calculation area in the treatment plan covers this device, the radiation attenuation caused by passing through the materials can be included in the calculation.

We plan to perform a load test in cooperation with healthy people and to develop special software to calculate the twist automatically. In this context, an important issue is that the cervical spine is known to have a coupling motion (axial rotation with lateral bending and vice versa).^13^ By quantifying the coupling motion of the cervical spine accompanying the movement of the prototype device, the software could have a program that takes into account the coupling motion. In addition, the connection with radiotherapy devices is one of important tasks for software development. Recently, a commercial system that can confirm the patient position by optical laser three‐dimensional surface imaging was released^14^, ^15^, ^16^; however, if the system detects twist of the patient, correction for this can only be done manually. Once our system can automatically and remotely be controlled (in development), the combination of the body surface detection system and our system might be able to correct the twist of the patient without touching the patient's body. In addition, we plan to support the thermoplastic mask on the HN and body plates to prevent patient movement during dose delivery. The applicable mask unit is a separate type divided into HN and shoulder parts. Without the mask, the combination with optical laser three‐dimensional surface imaging would be effective for the reduction of patient movement during dose delivery.

This feasibility study should serve as a proof of concept for future clinical implementation to reduce the residual error of the patient position and reduce the additional margin needed for compensating the adequate dose to tumors. However, our device has some limitations. First, our device still has some challenges for a clinical application as mentioned above. The mechanism for attaching the thermoplastic mask on the HN and body plates is not included, and special software for calculating the rotational angles and equipment for automatically correcting the angles are under development. Next, our device might not be suitable for cases with sufficient safety margin for the tumor. The efficacy of the device would be shown for the cases, wherein the accuracy of patient position is particularly required (e.g., IMRT for HN cancer and stereotactic radiotherapy for the recurrence). Finally, our device can be attached to the CT and treatment couches with commercial lock bars; therefore, the position reproducibility of the device is high. Conversely, the flatness of the patient needs to be maintained by setting a cushion on the patient’s back to fill the difference in level between the couch and the body plate of the device.

## 5 CONCLUSIONS

We developed a twist‐correction system for HN radiotherapy. We found that the rotational accuracy and repeatability exhibited good performance under no load. Our prototype device still has some challenges for a clinical application: the development of special software for calculating the rotational angles and equipment for automatically correcting the angles; the mechanism for attaching the thermoplastic mask on the HN and body plates. Conversely, the clinical application of this concept could be expected to significantly reduce the residual error in the patient position as well as reduce the additional margin required to compensate for the adequate dose to the tumor.

## CONFLICT OF INTEREST

No conflict of interest.
